# Racial and Ethnic Differences in Homicides of Adult Women and the Role of Intimate Partner Violence — United States, 2003–2014

**DOI:** 10.15585/mmwr.mm6628a1

**Published:** 2017-07-21

**Authors:** Emiko Petrosky, Janet M. Blair, Carter J. Betz, Katherine A. Fowler, Shane P.D. Jack, Bridget H. Lyons

**Affiliations:** 1 Division of Violence Prevention, National Center for Injury Prevention and Control, CDC.

Homicide is one of the leading causes of death for women aged ≤44 years.* In 2015, homicide caused the death of 3,519 girls and women in the United States. Rates of female homicide vary by race/ethnicity (*1*), and nearly half of victims are killed by a current or former male intimate partner (*2*). To inform homicide and intimate partner violence (IPV) prevention efforts, CDC analyzed homicide data from the National Violent Death Reporting System (NVDRS) among 10,018 women aged ≥18 years in 18 states during 2003–2014. The frequency of homicide by race/ethnicity and precipitating circumstances of homicides associated with and without IPV were examined. Non-Hispanic black and American Indian/Alaska Native women experienced the highest rates of homicide (4.4 and 4.3 per 100,000 population, respectively). Over half of all homicides (55.3%) were IPV-related; 11.2% of victims of IPV-related homicide experienced some form of violence in the month preceding their deaths, and argument and jealousy were common precipitating circumstances. Targeted IPV prevention programs for populations at disproportionate risk and enhanced access to intervention services for persons experiencing IPV are needed to reduce homicides among women.

CDC’s NVDRS is an active state-based surveillance system that monitors characteristics of violent deaths, including homicides. The system links three data sources (death certificates, coroner/medical examiner reports, and law enforcement reports) to create a comprehensive depiction of who dies from violence, where and when victims die, and factors perceived to contribute to the victim’s death (*3*). This report includes NVDRS data from 18 states during 2003–2014 (all available years).^†^ Five racial/ethnic categories^§^ were used for this analysis: white, black, American Indian/Alaska Native (AI/AN), Asian/Pacific Islander (A/PI), and Hispanic. Persons categorized as Hispanic might have been of any race. Persons categorized as one of the four racial populations were all non-Hispanic. Analyses were limited to female decedents aged ≥18 years. IPV-related deaths were defined as those involving intimate partner homicides (i.e., the victim was an intimate partner [e.g., current, former, or unspecified spouse or girlfriend] of the suspect), other deaths associated with IPV, including victims who were not the intimate partner (i.e., family, friends, others who intervened in IPV, first responders, or bystanders), or jealousy. Deaths where jealousy, such as in a lovers’ triangle, was noted as a factor were included only when they involved an actual relationship (versus unrequited interest). Violence experienced in the preceding month refers to all types of violence (e.g., robbery, assault, or IPV) that was distinct and occurred before the violence that killed the victim; there did not need to be any causal link between the earlier violence and the death itself (e.g., victim could have experienced a robbery by a stranger 2 weeks before being killed by her spouse).

Rates were calculated using intercensal and postcensal bridged–race population estimates compiled by CDC’s National Center for Health Statistics and were age-adjusted to the 2010 standard U.S. population of women aged ≥18 years (*4*). Sociodemographic characteristics and precipitating circumstances across racial/ethnic groups were examined using chi-square and Fisher’s exact tests. Two-sided p-values <0.05 were considered statistically significant. Differences in victim and incident characteristics by race/ethnicity were examined using chi-square and Fisher’s exact tests with posthoc pairwise comparisons of significant results; Bonferroni correction was applied to account for multiple comparisons.

From 2003 through 2014, a total of 10,018 female homicides were captured by NVDRS; among these, 1,835 (18.3%) were part of a homicide-suicide incident (i.e., suspect died by suicide after perpetrating homicide). Homicide victims ranged in age from 18 to 100 years. The overall age-adjusted homicide rate was 2.0 per 100,000 women. By race/ethnicity, non-Hispanic black women had the highest rate of dying by homicide (4.4 per 100,000), followed by AI/AN (4.3), Hispanic (1.8), non-Hispanic white (1.5), and A/PI women (1.2).

Approximately one third of female homicide victims (29.4%) were aged 18–29 years (Table 1); a larger proportion of non-Hispanic black and Hispanic victims were in this youngest age group than were non-Hispanic white and A/PI victims (p<0.01). The largest proportion of victims were never married or single at the time of death (38.2%); this proportion was highest among non-Hispanic black victims (59.2%; p<0.01). One third of victims had attended some college or more; history of college attendance was highest among non-Hispanic white (36.8%) and A/PI victims (46.2%; p<0.01). Approximately 15% of women of reproductive age (18–44 years) were pregnant or ≤6 weeks postpartum. Firearms were used in 53.9% of female homicides, most commonly among non-Hispanic black victims (57.7%; p<0.01). Sharp instrument (19.8%); hanging, suffocation, or strangulation (10.5%); and blunt instrument (7.9%) were other common mechanisms. Over half of all female homicides (55.3%) for which circumstances were known were IPV-related. A larger percentage of IPV-related female homicides were perpetrated by male suspects than were non-IPV-related homicides (98.2% versus 88.5%, respectively; p<0.01).

**TABLE 1:** Number and percentage* of homicides of females aged ≥18 years, by victim and incident characteristics — National Violent Death Reporting System, 18 states,^†^ 2003–2014

Characteristic	No. (%)					
	Total (N = 10,018)	White, non-Hispanic (n = 5,206)	Black, non-Hispanic (n = 3,514)	American Indian/Alaska Native (n = 240)	Asian/Pacific Islander (n = 236)	Hispanic^§^ (n = 822)
**Age group (yrs)**						
18–29^¶^	**2,947 (29.4)**	1,113 (21.4)**^,††,§§^	1,359 (38.7)^¶¶,^***	87 (36.3)^¶¶^	59 (25.0)**^,§§^	329 (40.0)^¶¶,^***
30–39^¶^	**2,179 (21.8)**	990 (19.0)**^,§§^	829 (23.6)^§§,¶¶^	56 (23.3)	59 (25.0)	245 (29.8)**^,¶¶^
40–49^¶^	**2,071 (20.7)**	1,126 (21.6)	704 (20.0)	52 (21.7)	46 (19.5)	143 (17.4)
50–59^¶^	**1,293 (12.9)**	824 (15.8)**^,§§^	352 (10.0)^¶¶^	25 (10.4)	31 (13.1)	61 (7.4)^¶¶^
≥60^¶^	**1,528 (15.3)**	1,153 (22.1)**^,††,§§^	270 (7.7)^¶¶,^***	20 (8.3)^¶¶,^***	41 (17.4)**^,††,§§^	44 (5.4)^¶¶,^***
**Marital status**						
Married, civil union, or domestic partnership^¶^	**3,156 (32.0)**	1,999 (38.9)**^,††,§§,^***	751 (21.9)^§§,¶¶,^***	51 (21.4)^¶¶,^***	121 (51.7)**^,††,§§,¶¶^	234 (28.7)**^,¶¶,^***
Never married or single^¶^	**3,766 (38.2)**	1,183 (23.0)**^,††,§§^	2,035 (59.2)^††,§§,¶¶,^***	118 (49.6)**^,¶¶,^***	52 (22.2)**^,††,§§^	378 (46.4)**^,¶¶,^***
Separated, divorced or widowed^¶^	**2,938 (29.8)**	1,954 (38.0)**^,††,§§,^***	651 (18.9)^††,§§,¶¶^	69 (29.0)**^,¶¶^	61 (26.1)^¶¶^	203 (24.9)**^,¶¶^
**Education^†††^**						
<High school graduate or GED equivalent^¶^	**2,143 (24.5)**	982 (21.2)**^,††,§§^	749 (25.6)^§§,¶¶^	75 (32.5)^¶¶,^***	39 (18.6)^††,§§^	298 (39.8)**^,¶¶,^***
High school graduate or GED equivalent^¶^	**3,672 (41.9)**	1,952 (42.1)	1,261 (43.0)	105 (45.5)	74 (35.2)	280 (37.4)
Some college or more^¶^	**2,946 (33.6)**	1,707 (36.8)**^,††,§§^	921 (31.4)^††,§§,¶¶,^***	51 (22.1)**^,¶¶,^***	97 (46.2)**^,††,§§^	170 (22.7)**^,¶¶,^***
**Pregnancy status^§§§^**						
Pregnant or ≤6 weeks postpartum^¶^	**298 (15.2)**	120 (12.9)**	134 (18.6)^¶¶^	7 (13.2)	6 (14.3)	31 (14.6)
**Method**						
Firearm^¶^	**5,234 (53.9)**	2,681 (53.4)**^,††,^***	1,975 (57.7)^††,§§,¶¶,^***	90 (38.8)**^,§§,¶¶^	92 (40.0)**^,¶¶^	396 (49.4)**^,††^
Sharp instrument^¶^	**1,918 (19.8)**	878 (17.5)**^,§§,^***	715 (20.9)^§§,¶¶,^***	49 (21.1)	70 (30.4)**^,¶¶^	206 (25.7)**^,¶¶^
Hanging, suffocation, strangulation^¶^	**1,017 (10.5)**	542 (10.8)	325 (9.5)^§§^	15 (6.5)	32 (13.9)	103 (12.9)**
Blunt instrument^¶^	**770 (7.9)**	453 (9.0)**^,††,§§^	216 (6.3)^††,¶¶^	40 (17.2)**^,§§,¶¶,^***	16 (7.0)^††^	45 (5.6)^††,¶¶^
Other (single method) ^¶^	**765 (7.9)**	467 (9.3)**^,††^	189 (5.5)^††,¶¶^	38 (16.4)**^,§§,¶¶^	20 (8.7)	51 (6.4)^††^
**IPV^¶¶¶^**						
IPV-related^¶,^****	**4,442 (55.3)**	2,446 (56.8)**	1,360 (51.3)^§§,¶¶^	112 (55.4)	118 (57.8)	406 (61.0)**

**Abbreviations:** GED = General Education Development; IPV = intimate partner violence.

* Excludes decedents with missing, unknown, and other race/ethnicity (n = 61). Percentages might not sum to 100% because of rounding.

^†^ Alaska, Colorado, Georgia, Kentucky, Maryland, Massachusetts, Michigan, New Jersey, New Mexico, North Carolina, Ohio, Oklahoma, Oregon, Rhode Island, South Carolina, Utah, Virginia, and Wisconsin.

^§^ Includes persons of any race.

^¶^ Characteristic with a statistically significant result.

** Significantly different from non-Hispanic black females.

^††^ Significantly different from American Indian/Alaska Native females.

^§§^ Significantly different from Hispanic females.

^¶¶^ Significantly different from non-Hispanic white females.

*** Significantly different from Asian/Pacific Islander females.

^†††^ “<High school graduate/GED equivalent” includes 11th grade and below. “High school graduate/GED equivalent” includes 12th grade. “Some college or more” includes some college credit, associate’s degree, master’s degree, doctorate, and professional degrees.

^§§§^ Includes only females of reproductive age (18–44 years) with known pregnancy status (n = 1,957).

^¶¶¶^ Includes only decedents where circumstances were known (n = 8,028).

**** Includes cases with victim-suspect relationship of intimate partner (current, former, or unspecified spouse or girlfriend), other deaths associated with IPV, or IPV-related jealousy/lovers’ triangle.

Circumstance information was known for all 4,442 IPV-related homicides and 3,586 (64.3%) non-IPV-related homicides and was examined further. Among IPV-related homicides, 79.2% and 14.3% were perpetrated by a current or former intimate partner, respectively (Table 2). Approximately one in 10 victims experienced some form of violence in the month preceding their death. However, only 11.2% of all IPV-related homicides were precipitated by another crime; 54.4% of these incidents involved another crime in progress. The most frequently reported other precipitating crimes were assault/homicide (45.6%), rape/sexual assault (11.1%), and burglary (9.9%). In 29.7% of IPV-related homicides, an argument preceded the victim’s death; this occurred more commonly among Hispanic victims than among non-Hispanic black and white victims. Approximately 12% of IPV-related homicides were associated with jealousy; this circumstance was also documented more commonly among Hispanic victims than among non-Hispanic black and white victims.

**TABLE 2:** Number and percentage* of homicides of females aged ≥18 years, by race/ethnicity, victim’s relationship to suspect, and precipitating circumstances^†^ for intimate partner violence (IPV)–related deaths — National Violent Death Reporting System, 18 states,**^§^** 2003–2014.

Characteristic	No. (%)					
	Total (N = 4,442)	White, non-Hispanic (n = 2,446)	Black, non-Hispanic (n = 1,360)	American Indian/Alaska Native (n = 112)	Asian/Pacific Islander (n = 118)	Hispanic^¶^ (n = 822)
Victim-suspect relationship**						
Current intimate^††^ partner	**3,417 (79.2)**	1,927 (81.0)^§§^	1,007 (76.6)^¶¶^	88 (81.5)	94 (81.0)	301 (75.8)
Former intimate partner^††^	**618 (14.3)**	322 (13.5)	198 (15.1)	13 (12.0)	11 (9.5)	74 (18.6)
Other^††,^***	**278 (6.4)**	129 (5.4)^§§^	109 (8.3)^¶¶^	7 (6.5)	11 (9.5)	22 (5.5)
**Circumstances**						
Victim experienced violence in the past month^†††^	**265 (11.2)**	147 (10.8)	66 (9.9)	10 (16.7)	9 (12.9)	33 (15.6)
Precipitated by another crime	**496 (11.2)**	261 (10.7)	166 (12.2)	10 (8.9)	13 (11.0)	46 (11.3)
Crime in progress^§§§^	**270 (54.4)**	137 (52.5)	93 (56.0)	7 (70.0)	7 (53.8)	26 (56.5)
Argument preceded victim's death^††^	**1,320 (29.7)**	660 (27.0)^¶¶¶^	420 (30.9)^¶¶¶^	36 (32.1)	42 (35.6)	162 (39.9)^§§,¶¶^
Jealousy/Lovers' triangle^††^	**516 (11.6)**	262 (10.7)^¶¶¶^	143 (10.5)^¶¶¶^	21 (18.8)	13 (11.0)	77 (19.0)^§§,¶¶^

* Includes only decedents with one or more circumstances present: n = 4,442 (100%) IPV-related female homicides.

^†^ The sum of percentages in columns exceeds 100% because more than one circumstance could have been present per decedent.

^§^ Alaska, Colorado, Georgia, Kentucky, Maryland, Massachusetts, Michigan, New Jersey, New Mexico, North Carolina, Ohio, Oklahoma, Oregon, Rhode Island, South Carolina, Utah, Virginia, and Wisconsin.

^¶^ Includes persons of any race.

** Victim-suspect relationship known for 4,313 (97.1%) IPV-related female homicides.

^††^ Characteristic with statistically significant results.

^§§^ Significantly different from non-Hispanic black females.

^¶¶^ Significantly different from non-Hispanic white females.

*** Includes nonintimate partner victims of IPV-related female homicide (e.g., friend, family member, etc.).

^†††^ Variable collected for homicides since 2009. Denominator is IPV-related female homicides during 2009–2014 (n = 2,369).

^§§§^ Denominator includes only those decedents involved in an incident that was precipitated by another crime.

^¶¶¶^ Significantly different from Hispanic females.

Among non-IPV related female homicides with known suspects, the victim’s relationship to the suspect was most often that of acquaintance (19.7%), stranger (15.7%), another person known to the victim in which the exact nature of the relationship or prior interaction was unclear (15.2%), or parent (15.2%) (Table 3). Non-Hispanic black victims were significantly more likely to be killed by an acquaintance (29.0%) than were non-Hispanic white victims (14.9%). A/PI and Hispanic victims were significantly more likely to be killed by a stranger (28.6% and 24.1%, respectively) than were non-Hispanic white victims (13.9%). Fewer than 2% of non-IPV related homicide victims experienced violence during the preceding month (data not shown). However, a substantial percentage of these homicides (41.6%) were precipitated by another crime; 67.2% of these incidents involved another crime in progress. The type of other precipitating crime was most frequently robbery (31.1%), assault/homicide (21.3%), burglary (12.2%), or rape/sexual assault (11.2%). Female homicides involving A/PI victims were more likely to be precipitated by another crime (57.0%) than were homicides involving non-Hispanic black (40.7%) and Hispanic (35.4%) victims. In 37.8% of non-IPV related homicides, an argument preceded the victim’s death, more commonly among AI/AN (47.8%) and non-Hispanic black (41.1%) victims than among A/PI (25.6%) victims.

**TABLE 3:** Number and percentage* of homicides of females aged ≥18 years, by race/ethnicity, victim’s relationship to suspect and precipitating circumstances**^†^** for nonintimate partner violence (IPV)–related deaths — National Violent Death Reporting System, 18 states,**^§^** 2003–2014.

Characteristic	No. (%)					
	Total (N = 3,586)	White, non-Hispanic (n = 1,859)	Black, non-Hispanic (n = 1,291)	American Indian/Alaska Native (n = 90)	Asian/Pacific Islander (n = 86)	Hispanic^¶^ (n = 260)
Victim-suspect relationship**						
Acquaintance^††^	**439 (19.7)**	188 (14.9)^§§^	190 (29.0)^¶¶^	16 (24.2)	9 (14.3)	36 (20.7)
Stranger^††^	**349 (15.7)**	176 (13.9)***^,†††^	103 (15.7)	10 (15.2)	18 (28.6)^¶¶^	42 (24.1)^¶¶^
Other person, known to victim	**339 (15.2)**	195 (15.4)	103 (15.7)	9 (13.6)	8 (12.7)	24 (13.8)
Parent^††^	**337 (15.2)**	237 (18.7)^§§,†††^	79 (12.0)^¶¶^	4 (6.1)	7 (11.1)	10 (5.7)^¶¶^
Other^††^	**760 (34.2)**	469 (37.1)^§§^	181 (27.6)^¶¶^	27 (40.9)	21 (33.3)	62 (35.6)
**Circumstances**						
Precipitated by another crime^††^	**1,492 (41.6)**	788 (42.4)	526 (40.7)***	37 (41.1)	49 (57.0)^§§,†††^	92 (35.4)***
Crime in progress^§§§^	**1,002 (67.2)**	535 (67.9)	345 (65.6)	25 (67.6)	33 (67.3)	64 (69.6)
Argument preceded victim's death^††^	**1,357 (37.8)**	659 (35.4)^§§^	531 (41.1)^¶¶,^***	43 (47.8)***	22 (25.6)^§§,¶¶¶^	102 (39.2)

* Denominator includes only decedents with one or more circumstances present: n = 3,586 (64.3%) non-IPV related homicides.

^†^ The sum of percentages in columns exceeds 100% because more than one circumstance could have been present per decedent.

^§^ Alaska, Colorado, Georgia, Kentucky, Maryland, Massachusetts, Michigan, New Jersey, New Mexico, North Carolina, Ohio, Oklahoma, Oregon, Rhode Island, South Carolina, Utah, Virginia, and Wisconsin.

^¶^ Includes persons of any race.

** Victim-suspect relationship known for 2,224 (62.0%) non-IPV-related female homicide victims.

^††^ Characteristic with a statistically significant result.

^§§^ Significantly different from non-Hispanic black females.

^¶¶^ Significantly different from non-Hispanic white females.

*** Significantly different from Asian/Pacific Islander females.

^†††^ Significantly different from Hispanic females.

^§§§^ Denominator includes only those decedents involved in an incident that was precipitated by another crime.

^¶¶¶^ Significantly different from American Indian/Alaska Native females.

## Discussion

Homicide is the most severe health outcome of violence against women. Findings from this study of female homicides from NVDRS during 2003–2014 indicate that young women, particularly racial/ethnic minority women, were disproportionately affected. Across all racial/ethnic groups of women, over half of female homicides for which circumstances were known were IPV-related, with >90% of these women being killed by their current or former intimate partner.

Strategies to prevent IPV-related homicides range from protecting women from immediate harm and intervening in current IPV, to developing and implementing programs and policies to prevent IPV from occurring (*5*). IPV lethality risk assessments conducted by first responders have shown high sensitivity in identifying victims at risk for future violence and homicide (*6*). These assessments might be used to facilitate immediate safety planning and to connect women with other services, such as crisis intervention and counseling, housing, medical and legal advocacy, and access to other community resources (*6*). State statutes limiting access to firearms for persons under a domestic violence restraining order can serve as another preventive measure associated with reduced risk for intimate partner homicide and firearm intimate partner homicide (*7*). Approximately one in 10 victims of IPV-related homicide experienced some form of violence in the preceding month, which could have provided opportunities for intervention. Bystander programs, such as Green Dot,^¶^ teach participants how to recognize situations or behaviors that might become violent and safely and effectively intervene to reduce the likelihood of assault (*8*). In health care settings, the U.S. Preventive Services Task Force recommends screening women of childbearing age for IPV and referring women who screen positive for intervention services.** Approximately 15% of female homicide victims of reproductive age (18–44 years) were pregnant or postpartum, which might or might not be higher than estimates in the general U.S. female population, requiring further examination.

Approximately 40% of non-Hispanic black, AI/AN, and Hispanic female homicide victims were aged 18–29 years. Argument and jealousy were common precipitating factors for IPV-related homicides. Teaching safe and healthy relationship skills is an important primary prevention strategy with evidence of effectiveness in reducing IPV by helping young persons manage emotions and relationship conflicts and improve their problem-solving and communication skills (*5*). Preventing IPV also requires addressing the community- and system-level factors that increase the risk for IPV; neighborhoods with high disorder, disadvantage, and poverty, and low social cohesion are associated with increased risk of IPV (*5*), and underlying health inequities caused by barriers in language, geography, and cultural familiarity might contribute to homicides, particularly among racial/ethnic minority women (*9*).

The findings in this report are subject to at least five limitations. First, NVDRS data are available from a limited number of states and are therefore not nationally representative. Second, race/ethnicity data on death certificates might be misclassified, particularly for Hispanics, A/PI, and AI/AN (*10*). Third, the female homicide victims in this dataset were more likely to be never married or single and less likely to have attended college than the general U.S. female population^††^; although this is likely attributable to the relatively younger age distribution of homicide victims in general,^§§^ this requires further examination. Fourth, not all homicide cases include detailed suspect information; in this analysis, 85.3% of cases included information on the suspect. Finally, information about male corollary victims of IPV-related homicide (i.e., other deaths associated with IPV, including male victims who were not the intimate partner) were not included in this analysis. Therefore, the full scope of IPV-related homicides involving women is not captured.

The racial/ethnic differences in female homicide underscore the importance of targeting prevention and intervention efforts to populations at disproportionately high risk. Addressing violence will require an integrated response that considers the influence of larger community and societal factors that make violence more likely to occur.

**Summary**

**What is already known about this topic?**

Homicide is one of the leading causes of death for women aged ≤44 years, and rates vary by race/ethnicity. Nearly half of female victims are killed by a current or former male intimate partner.

**What is added by this report?**

Homicides occur in women of all ages and among all races/ethnicities, but young, racial/ethnic minority women are disproportionately affected. Over half of female homicides for which circumstances were known were related to intimate partner violence (IPV). Arguments and jealousy were common precipitating circumstances among IPV-related homicides. One in 10 victims of IPV-related homicide were reported to have experienced violence in the month preceding their deaths.

**What are the implications for public health practice?**

Racial/ethnic differences in female homicide underscore the importance of targeting intervention efforts to populations at risk and the conditions that increase the risk for violence. IPV lethality risk assessments might be useful tools for first responders to identify women at risk for future violence and connect them with life-saving safety planning and services. Teaching young persons safe and healthy relationship skills as well as how to recognize situations or behaviors that might become violent are effective IPV primary prevention measures.

## Footnotes

* CDC’s Web-based Injury Statistics Query and Reporting System (WISQARS). https://www.cdc.gov/injury/wisqars/index.html.

† In 2003, the National Violent Death Reporting System (NVDRS) began data collection with six states (Maryland, Massachusetts, New Jersey, Oregon, South Carolina, and Virginia) participating; seven states (Alaska, Colorado, Georgia, North Carolina, Oklahoma, Rhode Island, and Wisconsin) joined in 2004, four (California, Kentucky, New Mexico, and Utah) in 2005, and two (Ohio and Michigan) in 2010. California did not collect statewide data and concluded participation in 2009. Ohio collected statewide data starting in 2011 and Michigan starting in 2014. CDC provides funding for state participation, and the ultimate goal is for NVDRS to expand to include all 50 states, U.S. territories, and the District of Columbia.

§ Information on race and ethnicity are recorded as separate items in NVDRS consistent with U.S. Department of Health and Human Services (HHS) and Office of Management and Budget standards for race/ethnicity categorization. HHS guidance on race/ethnicity is available at https://aspe.hhs.gov/datacncl/standards/ACA/4302/index.shtml.

¶ http://www.livethegreendot.com.

** https://www.uspreventiveservicestaskforce.org/Page/Document/RecommendationStatementFinal/intimate-partner-violence-and-abuse-of-elderly-and-vulnerable-adults-screening.

†† https://www.census.gov/acs/www/data/data-tables-and-tools/.

§§ https://www.cdc.gov/nchs/data/nvsr/nvsr65/nvsr65_04.pdf.
